# Anchor and Krackow‐“8” Suture for the Fixation of Distal Pole Fractures of the Patella: Comparison to Kirschner Wire

**DOI:** 10.1111/os.13124

**Published:** 2021-12-29

**Authors:** Jia Xie, Yu Fu, Jun Li, Hao Yu, Yong Zhang, Juehua Jing

**Affiliations:** ^1^ Department of Orthopedics The Second Hospital of Anhui Medical University Hefei China; ^2^ Department of Orthopedics, Hefei Cancer Hospital Chinese Academy of Sciences Hefei China

**Keywords:** Anchor suture, Distal pole patellar, Fracture, Kirschner wire, Krackow‐“8” suture fixation

## Abstract

**Objective:**

The study aim was to evaluate the clinical outcomes, functional outcomes, and postoperative complications of anchor and Krackow‐“8” suture fixation (AS) and K‐wire fixation in patients with distal pole patellar fractures.

**Methods:**

Twenty‐eight patients with distal pole patella fractures between January 2011 and December 2014 were reviewed retrospectively. The anchor and Krackow‐“8” suture fixation (AS group) was applied in 10 patients and 18 patients underwent K‐wire fixation (K‐wire group). The average age of patients was 46.000 ± 19.476 years in the AS group and 47.556 ± 15.704 years in the K‐wire group, with comparable demographic characteristics. All patients underwent regular follow‐up the operative data and postoperative functional and clinical outcomes were recorded. Complications were recorded by clinical and radiographic assessment. Bostman patellar fracture functional score was used to evaluate knee function after patellar fracture.

**Results:**

A total of 28 eligible patients were included in this study. The mean follow‐up was similar for the AS and the K‐wire groups (P > 0.05). The incision length of AS group was significantly smaller than that of K‐wire group (P < 0.05). The incision length of AS group was significantly smaller than that of K‐wire group (P < 0.05). The final follow‐up on the range of motion of the knee: the average extension lag was similar in two groups (P > 0.05); flexion and flexion–extension angle was slightly better in the AS group than in the K‐wire group. The Bostman patella fracture functional score of AS group were better than K‐wire group at 3 and 6 months after operation. Four kinds of postoperative complications in two groups, one patient (10%) in the AS group and two patients (11.1%) in the K‐wire group had infections. Two (11.1%) cases of nonunion in group K and three patients (16.7%) required re‐operation: one due to infection and two due to early implant failure. In the AS group, all distal pole fractures of the patella showed bony union, without loosening, falling, pulling out and nonunion of the fractures 6 months after operation.

**Conclusions:**

Anchor and Krackow‐“8” suture fixation is an easily executed surgical procedure that can significantly reduce incision length and achieve better surgical outcomes than traditional procedures with regard to postoperative complications, knee function and without requiring a second operation. This technique is an effective operation method for the treatment of inferior patellar pole fractures.

## Graphical abstract

The study demonstrates that AS is an easily executed surgical procedure that can significantly reduce incision length and achieve better surgical outcomes compared to the traditional technique with regard to postoperative complications, knee function and without the need for a second operation.

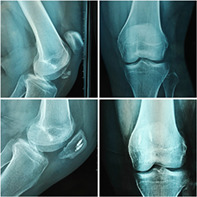

## Introduction

In most fracture classification systems, distal pole avulsion fractures of the patella fall into a separate category^1^, ^2^, ^3^. Such fractures account for 9.3% to 22.4% of all patellar fractures and are treated surgically if displaced or associated with complete disruption of the extensor mechanism^4^.

Patients with distal pole fractures of the patella have a disrupted extensor mechanism, which results in considerable functional disability. These fractures, particularly multi‐fragmentary fractures, are difficult to treat, and reconstruction with preservation of the inferior patellar pole with a normal height of the patella is sometimes impossible to achieve with standard techniques. The ideal method should comply with three crucial demands: it should aid in reduction of the fracture, provide stable fixation and enable early rehabilitation^5^.

Various methods have been introduced to fix distal patellar pole fractures, including tension band wiring, separate vertical wiring, the use of a basket plate, wiring through cannulated screws, and partial patellectomy, and each of these techniques has characteristic advantages and disadvantages. Currently, clinical and biomechanical studies have provided definitive evidence that resection disrupts the extensor mechanism by decreasing the lever arm at the knee joint^6^, ^7^. Operative fixation of displaced patella fractures has now become the standard of care for these injuries^8^. The modified anterior tension band technique using Kirschner wire (K‐wire) is one of the most common methods used for the fixation of inferior patellar pole fractures. Although the K‐wire and tension band technique remains popular, patients frequently complain of discomfort secondary to prominent hardware, leading to high rates of removal of hardware (ROH). Thus, revision surgery with K‐wire removal becomes necessary in up to 65% of cases^9^.

A novel technique that employs the application of two or three anchors to the patella and reattachment of the distal fragments together with the patellar tendon has recently been described^10^. Anchor suturing (AS) had previously been reported as a technique for patellar tendon rupture fixation and was compared to transosseous tunnel suturing^11^, ^12^, ^13^. All these studies agree that the strength of anchors, however, is inferior to that of intraosseous sutures but is apparently sufficient for fracture fixation and early mobilization^14^.

This study aims to quantify clinical and postoperative functional outcomes and to identify postoperative complications in a cohort of patients who were treated with non‐absorbable braided suture fixation for distal pole fractures of the patella. These patients were then compared to a control group of patients who were treated for distal pole fractures of the patella using Kirschner wire. We hypothesized that there would be no observable difference in outcomes between the two groups.

## Patients and Methods

The data used in this study were collected as part of a larger project that included all patients who were admitted to our department for the treatment of patellar fractures between January 2011 and December 2014. Our hospital's electronic medical record system was searched for patients who were admitted and operatively treated for patellar fractures. We searched the ICD‐9 codes for patellar fractures (822.0) and for patellar fracture‐related surgical procedure codes (77.86, 78.56, 79.36.04 and sub‐codes). A list of 251 patients was generated, of whom 223 underwent surgery.

Patients' demographic and operation information was obtained by manually reviewing their files. Since the intervals between patients' follow‐up visits were not constant, we were able to describe the range of knee motion at the last outpatient visit to our medical institute. Radiographs were reviewed by a single author using the picture archiving and communication system. Pre‐operative radiographs were reviewed to determine fracture type, and postoperative radiographs were reviewed for the type of fixation used and signs of fracture union. Fractures were described according to the OTA^15^ system and the more commonly used descriptive classification. The latter classification includes seven fracture patterns: nondisplaced, transverse, distal or proximal pole, multi‐fragmented nondisplaced, multi‐fragmented displaced, vertical and osteochondral^16^.

### Inclusion and Exclusion Criteria

According to different methods of fracture fixation, all patients had been divided K‐wire group and AS group. Informed consent was obtained from all patients.

Inclusion criteria: (i) diagnosed as distal pole avulsion fractures of the patella on CT or X‐rays; (ii) accepted anchor and Krackow‐“8” suture fixation (AS) and K‐wire fixation; (iii) complete follow‐up data; and (iv) retrospective study.

Excluded criteria: (i) patients with other types of patella fractures; (ii) open fractures; (iii) no history of surgery in the affected; and (iv) patients who were unable to understand the items on the questionnaire.

### Operation Techniques

#### Anesthesia and Position

All patients followed a standardized anesthesia regimen. With general anesthesia, the endotracheal tube should be attached to the opposite side so as not to interfere with the surgical area. After satisfactory anesthesia, the patient was placed in the supine position, surgical incision was prepared and draped and applied sterile tape.

#### Approach and Exposure

An anterior midline incision was made over the patella to the tibial tuberosity and incision was about 6cm. Along the incision line, the skin, subcutaneous tissue and deep fascia were cut in turn and the flap was removed upward to expose the patella.

#### Fixation or Placement of Prosthesis

**Fig. 1:**
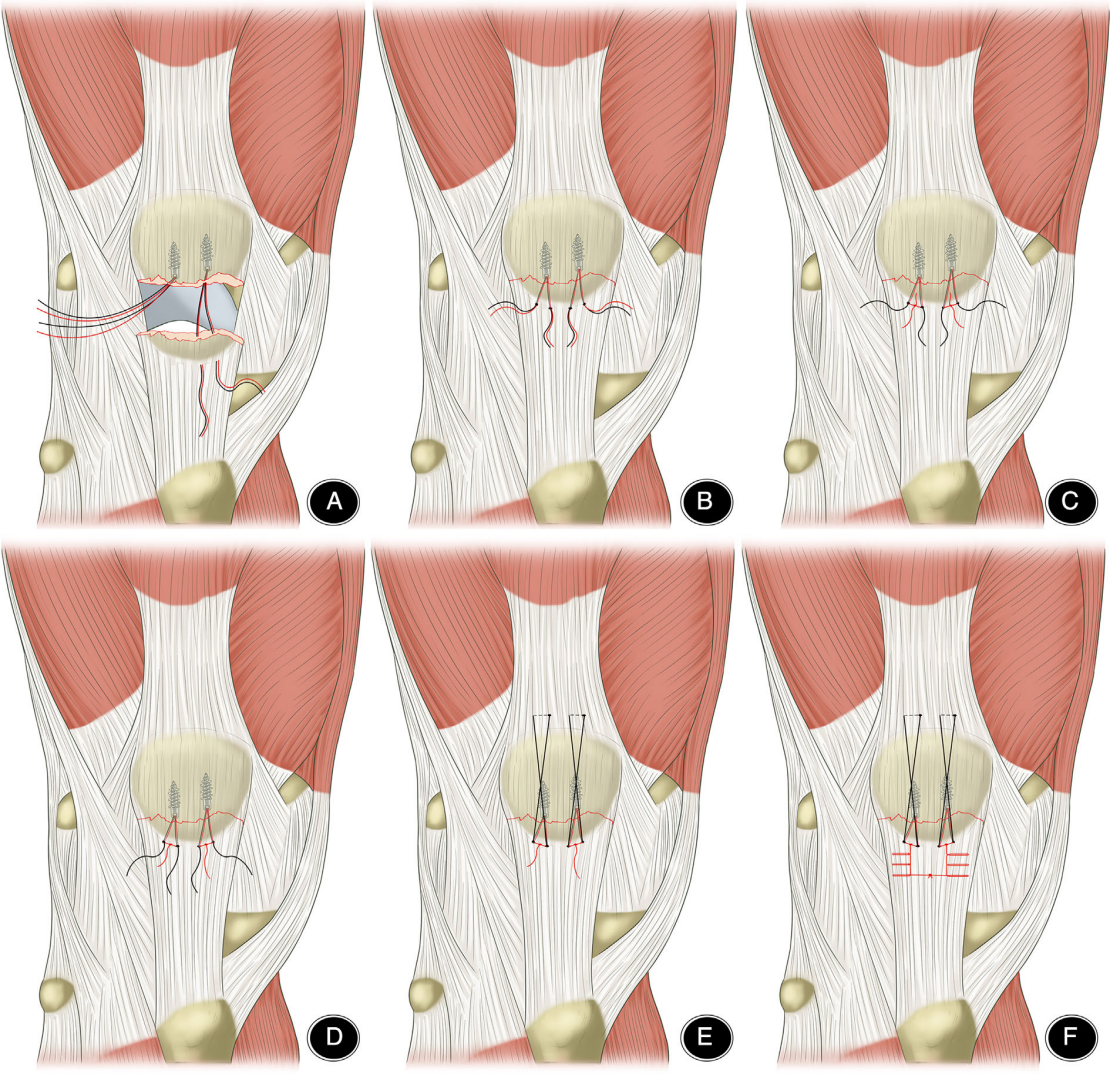
Anchor and Krackow‐“8” suture fixation in distal pole fractures of the patella. (A) Three equal portions of proximal patellar fracture, with anchors placed in the decile points. (B) Four equal portions distal to the fracture, with the Ultrabraid suture running through the corresponding point. (C,D). The use of Tauting wire to replace fracture reduction. (E) Ultrabraid sutures were sutured using “8” sutures over the patellar surface. (F) The distal patellar fragment and the patellar tendon are sutured using Krakow technique sutures.

Cleaned the blood clots in the broken end of the fracture and the joint capsule, check whether the articular surface is flat, and use the reduction forceps to clamp the reduction. Two suture anchors (TwinFix Ti 5.0 mm, Smith Nephew, London, UK) and Krackow‐“8” suture were inserted in the proximal patellar fracture fragment (Fig. 1). Three equal portions of proximal patellar fracture, with anchors placed in the decile points. Four equal portions distal to the fracture, with the Ultrabraid suture running through the corresponding point. The use of Tauting wire to replace fracture reduction. Ultrabraid sutures were sutured using “8” sutures over the patellar surface. The distal patellar fragment and the patellar tendon are sutured using Krakow technique sutures.

#### Postoperative Management

All patients were immobilized with a straight knee brace or cast, and postoperative functional exercise was directed by the surgeon. Appropriate knee flexion and extension exercise was performed 2 days postoperatively, active joint flexion and extension exercises 2 days postoperatively. Weeks 1 to 6 post‐surgery, gradual transition from partial to full weight bearing was performed under proper protection, and then physical therapy is performed gradually to restore full range of motion of the knee.

### Outcome Measures

#### Incision Length

The incision length was recorded for a general assessment of surgical complexity and degree of surgical trauma.

#### Range of Motion of Knee Joint

Measured the range of motion of the patient's knee at the last follow‐up, including extension degree, flexion degree and flexion–extension, which can be measured using a goniometer. Prolonged fixation of the knee joint will lead to the reduction of range of motion, which is related to the time and effect of postoperative rehabilitation.

#### Bostman Patellar Fracture Functional Score

Bostman patellar fracture functional score was used to evaluate knee function after patellar fracture. Bostman score system mainly includes eight aspects as range of motion (score 0–6), pain (score 0–6), work (score 0–4), atrophy (10 cm proximal of the patella) (score 0–4), assistive device (score 0–4), hydrops (score 0–2), soft leg symptoms (score 0–2) and climbing stairs (score 0–2). A total score < 20 is considered a fair score, 20–27 is a good and 28–30 excellent.

### Complications

Potential postoperative complications, as an indicator of surgical safety, include infection, nonunion, implant failure, and reoperation. All complications were recorded. The occurrence of complications affects the safety and feasibility of the operation.

### Statistical Analysis

The data were analyzed using SPSS for Windows version 17.0 (IBM, Armonk, NY, USA). Means and standard deviations (SDs) were used to describe continuous variables, and categorical variables are presented as numbers (percentages). Univariate analyses were performed using variance analysis for categorical data and Levene's‐test for continuous variables. The level of significance was set at *P* < 0.05.

## Results

### Operative Data

The demographic data of the 28 patients included in this study are presented in Table 1. All patients were followed up and responded to the questionnaire. Average follow‐up was 7.900 ± 2.283 months (range, 6 ‐ 12 months) in the AS group and 10.278 ± 4.212 months (range, 6 ‐ 12 months, *P* > 0.05) in the K‐wire group. Mean time from injury to surgery (h) was 9.300 ± 3.234 h (range, 6 ‐ 13 h) in the AS group and 9.111 ± 4.549 h (range, 4 ‐ 14 h) in the K‐wire group (*P* > 0.05).

**TABLE 1:** Comparisons of Anchor and Krackow‐“8” suture fixation (AS) to Kirschner wire (K‐wire) operating techniques for distal pole patella fractures (Mean ± SD†)

Variables	AS (n = 10)	K‐wire (n = 18)	*P* value
Age (years)	46.000 ± 19.476	47.556 ± 15.704	0.819
Follow‐up (months)	7.900 ± 2.283	10.278 ± 4.212	0.064
Time from injury to surgery (days)	9.300 ± 3.234	9.111 ± 4.549	0.909
Incision length (cm)	5.350 ± 0.851	11.722 ± 1.602	<0.05
Extension degree	3.000 ± 3.496	4.167 ± 3.930	0.442
Flexion degree	115.000 ± 16.499	100.833 ± 12.632	0.017
Flexion–extension	112.000 ± 17.826	96.667 ± 11.757	0.011
Bostman 3	27.100 ± 2.885	20.333 ± 1.495	<0.05
Bostman 6	28.300 ± 2.214	24.667 ± 1.970	<0.05

AS, Anchor and Krackow‐“8” suture fixation; K‐wire, Kirschner wire.

† Data represent numbers of patients and mean ± standard deviation.

Bostman3: Bostman patella fracture functional score at the third month of follow up. Bostman6: Bostman patella fracture functional score at the sixth month of follow up.

### Incision Length

Mean incision length was 5.350 ± 0.851 cm (range, 4 ‐ 7 cm) in the AS group and 11.722 ± 1.602 cm (range, 9 ‐ 14 cm) in the K‐wire group (*P* < 0.05).

### Range of Motion of Knee Joint

The patients' functional scores are reported in Table 1. Ranges of knee motion as examined at latest follow up in the clinic were similar between groups with an average extension lag of 3.000 ± 3.496 (range, 0 ‐ 10) degrees in the AS group compared with 4.167 ± 3.930 (range, 0 ‐ 10) degrees in the K‐wire group, and the difference did not reach significance (*P* = 0.442). However, knee flexion was 115.000 ± 16.499 (range, 95 ‐ 135) degrees in the AS group compared with 100.833 ± 12.632 (range, 85 ‐ 115) degrees in the K‐wire group (*P* = 0.017). The average flexion‐extension range was 112.000 ± 17.826 (range, 90 ‐ 135) degrees in the AS group compared with 96.667 ± 11.757 (range, 85 ‐ 115) degrees in the K‐wire group (*P* = 0.011).

### Bostman Patellar Fracture Functional Score

**Fig. 2:**
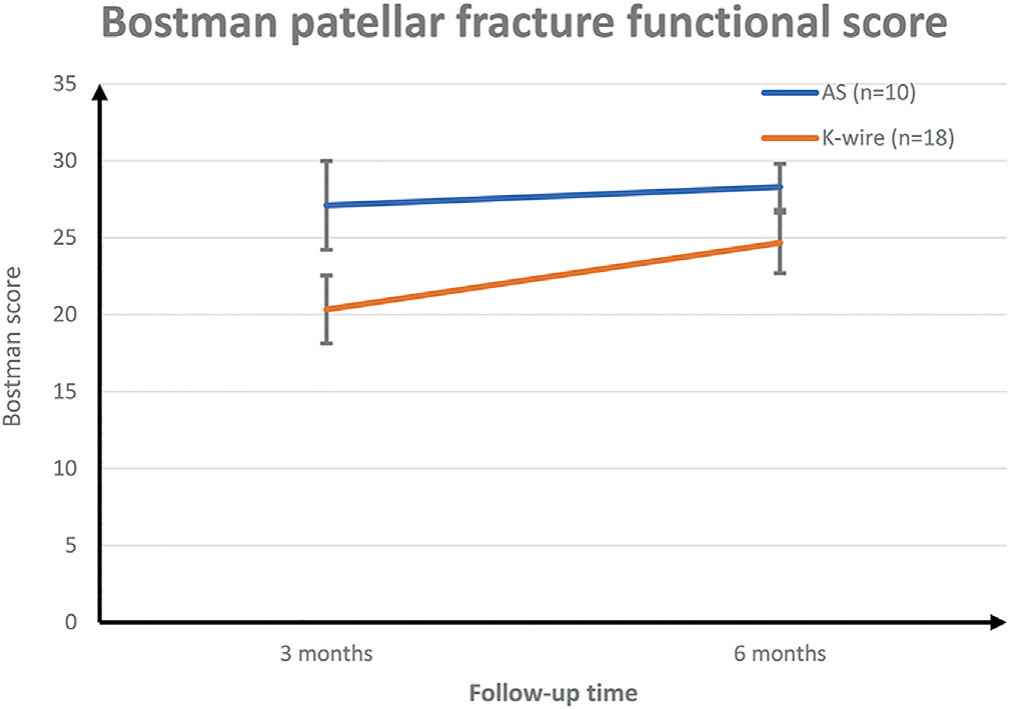
Bostman patellar fracture functional score.

The third month after surgery, the Bostman patella fracture functional score was 27.100 ± 2.885 (range, 22 ‐ 30) points in the AS group and 20.333 ± 1.495 (range, 18 ‐ 22) points in the K‐wire group (*P* < 0.05). The sixth month after surgery, the Bostman patella fracture functional score was 28.300 ± 2.214 (range, 23 ‐ 30) points in the AS group and 24.667 ± 1.970 (range, 21 ‐ 27) points in the K‐wire group (*P* < 0.05). (Fig. 2).

### Complications

**Fig. 3:**
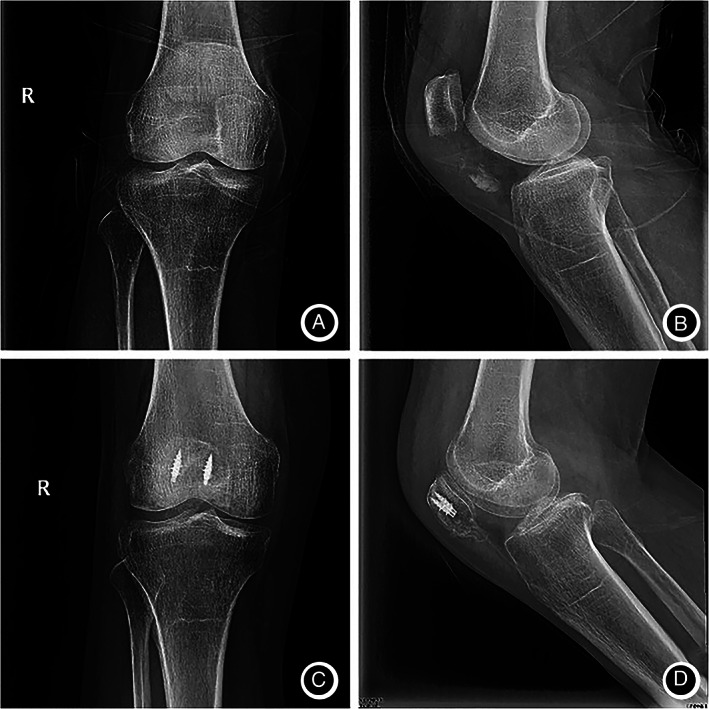
Anterioposterior and lateral knee radiographs of a 65‐year‐old female patient. (A,B) Patients with distal pole patellar fractures; (C,D) after treatment with anchor and Krackow‐“8” suture fixation.

**Fig. 4:**
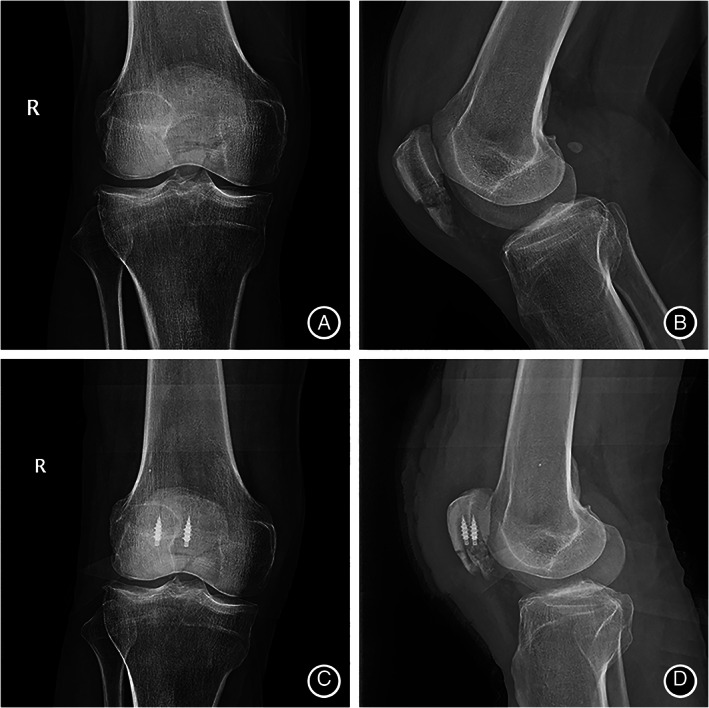
Anterioposterior and lateral knee radiographs of a 52‐year‐old male patient (A,B) Patients with distal pole patellar fractures; (C,D) after treatment with anchor and Krackow‐“8” suture fixation.

**Fig. 5:**
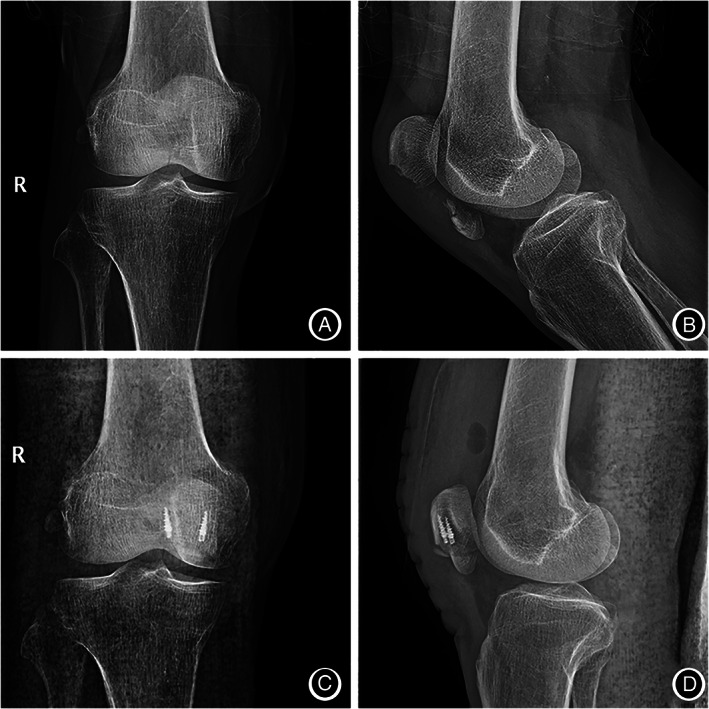
Anterioposterior and lateral knee radiographs of a 55‐year‐old male patient (A,B) Patients with distal pole patellar fractures; (C,D) after treatment with anchor and Krackow‐“8” suture fixation.

There were four postoperative complications due to surgical technique (Table 2). Three patients (10.7%) developed wound infection. However, one patient in the AS group had only one infection, whereas two patients in the K‐wire group had one infection. In the one case in the AS group, the wound was red and swollen, and the healing time was delayed because of open fracture. The wound healed after one debridement, and the other nine cases healed well. In contrast, two of the K‐wire patients (11.1%) had wound infection: one patient required only one debridement, and the other had a concomitant deep infection and early implant failure requiring complete serial debridements and re‐operation. Three patients (16.7%) required re‐operation in the K‐wire group: one due to infection and two due to early implant failure. In the AS group, all distal pole fractures of the patella showed bony union, without loosening, falling, pulling out and nonunion of the fractures 6 months after operation. The complication rate was lower in the AS group than that in the K‐wire group (*P* < 0.05). A typical case is shown in Figs 3, 4, 5.

**TABLE 2:** Postoperative complications according to surgical technique

Technique	n	Infection n (%)	Nonunion n (%)	Implant failure n (%)	Reoperation sn (%)
AS	10	1 (10%)	0	0	0
K‐wire	18	2 (11.1%)	2 (11.1%)	3 (16.7%)	3 (16.7%)
Total	28	3 (10.7%)	2 (7.1%)	3 (10.7%)	3 (10.7%)

AS, Anchor and Krackow‐“8” suture fixation; K‐wire, Kirschner wire.

## Discussion

Distal pole fractures of the patella are rare and are usually the result of direct trauma, with or without quadriceps muscle contraction. Although there are numerous reports on the subject, the choice of treatment for fractures of the inferior pole of the patella remains controversial. The complexity of the fracture usually precludes reconstruction; hence, some surgeons perform partial patellectomy as a last resort. However, partial patellectomy may result in an abnormal height of the patella and a significant loss of range of motion^17^.

### Previous Techniques

Patients who suffer from distal pole fractures of the patella have limited options for fracture fixation. Prior studies on patella fracture fixation have reported reoperation rates of between 20% and 50% following the use of Kirschner wire^18^, ^19^, ^20^, ^21^. A recent study with a 6.5‐year mean follow‐up by LeBrun reported a rate of 56% in their cohort^22^. All hardware removals occurred in the K‐wire group, accounting for 93% of all reoperations in the cohort; the remaining reoperations were indicated due to treatment failure^23^. Furthermore, the study showed that patients requiring reoperation had a significantly restricted range of motion in their affected knee, which remained significant after the exclusion of patients receiving pole resection^23^.

### Novel Tension Band

However, anchor suture fixation is clinically acceptable and has been biomechanically verified^24^, ^25^. In our study, patients undergoing anchor suture fixation experienced fewer hardware‐related postoperative complications and achieved higher Bostman scores at one‐year follow‐up. The reoperation rate was four times higher for patients receiving K‐wire fixation as compared with anchor suture fixation. This result is consistent with other reports, which demonstrated an increased reoperation rate for patients receiving metal implants.Although a previous study investigated the use of #5 Ethibond anchor suture fixation over K‐wire fixation^18^, it did not employ quantitative methods to assess patient outcome. Our study made use of accepted patient outcome metrics to quantify any differences between the two groups, as well as a basic chart review. The aforementioned studies using rigorous outcome metrics, such as LeBrun *et al*. and Tian *et al*. did not include a cohort that was treated with sutures. Our study reports the outcomes of applying the AS technique for distal pole patellar fractures on the largest cohort reported thus far, for a longer follow‐up time (14 months) and is the first to compare the AS technique to K‐wire operative techniques employed in this setting. Our results showed that AS had comparable functional outcomes, range of motion, complications and re‐operation rates compared to K‐wire (Tables 1 and 2). Mean incision length was significantly shorter for AS compared to K‐wire (5.35 cm and 11.722 cm, respectively; *P* < 0.05). These outcomes should be interpreted in light of the shorter follow‐up time for the AS group compared to the K‐wire group; these follow‐up differences are important when comparing a novel technique to a traditional one.

The complication rate was high for both the AS and K‐wire approaches. Surgical site infection was the most common complication (10.7%) and required re‐operation in all three cases. The currently reported infection rates are 0 ‐ 5% for patellar fractures and 0 ~ 11% for open fractures^9^, ^26^. Anand *et al*. reported no complications for the AS technique. We cannot account for the relatively high rate of infection in the present study. On the other hand, we had no case of symptomatic implant requiring re‐operation to remove it, as is commonly reported for other novel distal pole fixation techniques, such as the basket plate^7^. The rates of re‐operation are generally lower for K‐wire and AS compared to traditional techniques, most likely because the low profile of the construct does not irritate the patellar tendon^22^. Implant failure occurred in three patients in the K‐wire group shortly after surgery. In these cases, the anchor was pulled out of the main patellar fragment, and revision surgery and partial patellectomy were required. This complication has not been reported for anchor suturing in patellar fractures to date.

### Limitations

This study has several limitations, which are related to its being a retrospective evaluation of a novel technique; such studies are often prone to author bias. Another weak point is the difference in follow‐up time between the intervention and control groups. We also used subjective outcome measure tools and could not provide long‐term physical examination findings of actual knee strength, ranges of knee motion or patellofemoral signs. The primary reason for the latter drawback was to increase compliance by not requesting the patient to return for evaluation. We note that the percentage of patients available for similar follow‐up purposes in other studies was as low as 50%. Additionally, we feel that the relatively high patient satisfaction reflected by the subjective questionnaire is well correlated with good range of knee motion at the latest follow up.

### Conclusion

The application of AS for patellar inferior pole fracture fixation is a recently introduced and reported, novel surgical technique. We report the results of this technique on the largest cohort to date and compare the findings to those obtained using the traditional surgically treatment technique involving K‐wire. We conclude that AS is an easily executed surgical procedure that can significantly reduce incision length and achieve better surgical outcomes compared to the traditional technique with regard to postoperative complications, knee function and without the need for a second operation. The potential disadvantages of this technique are the high rates of postoperative infection (10%) and potential early hardware failure in the form of anchor pull out from the main patellar fragment. AS also entails higher costs than traditional techniques, an issue that is beyond the scope of this investigation. Further clinical trials that are free of the drawbacks of the present investigation are warranted to guide therapeutic decisions and confirm our belief that AS is a viable option for distal pole patellar fracture fixation.

## Ethics Approval and Consent to Participate

The protocol was approved by the clinical trial ethics committee/institutional review board (IRB) at The Second Hospital of Anhui Medical University, China. Informed consent was not required because the images and medical records of the patients had already been obtained and were anonymous.

## Author Declaration

All authors listed meet the authorship criteria according to the latest guidelines of the International Committee of Medical Journal Editors, and that all authors are in agreement with the manuscript.

## Authors' Contributions

Jun Li and Juehua Jing designed the study, Jia Xie and Yu Fu wrote the manuscript. Hao Yu, Yong Zhang carried out the statistical analysis. All authors read and approved the final manuscript.

## Availability of Data and Materials

All data generated or analyzed during this study are included in this article.
